# Supplementary material for: Preharvest Sprouting in Quinoa: A New Screening Method Adapted to Panicles and GWAS Components
Source: Plants (Basel). 2024 May 8;13(10):1297. doi: 10.3390/plants13101297 (PMC11124833; doi:10.3390/plants13101297)
Supplement: Supplementary file 1 [file plants-13-01297-s001.zip › Quinoa Figure S1.pdf]

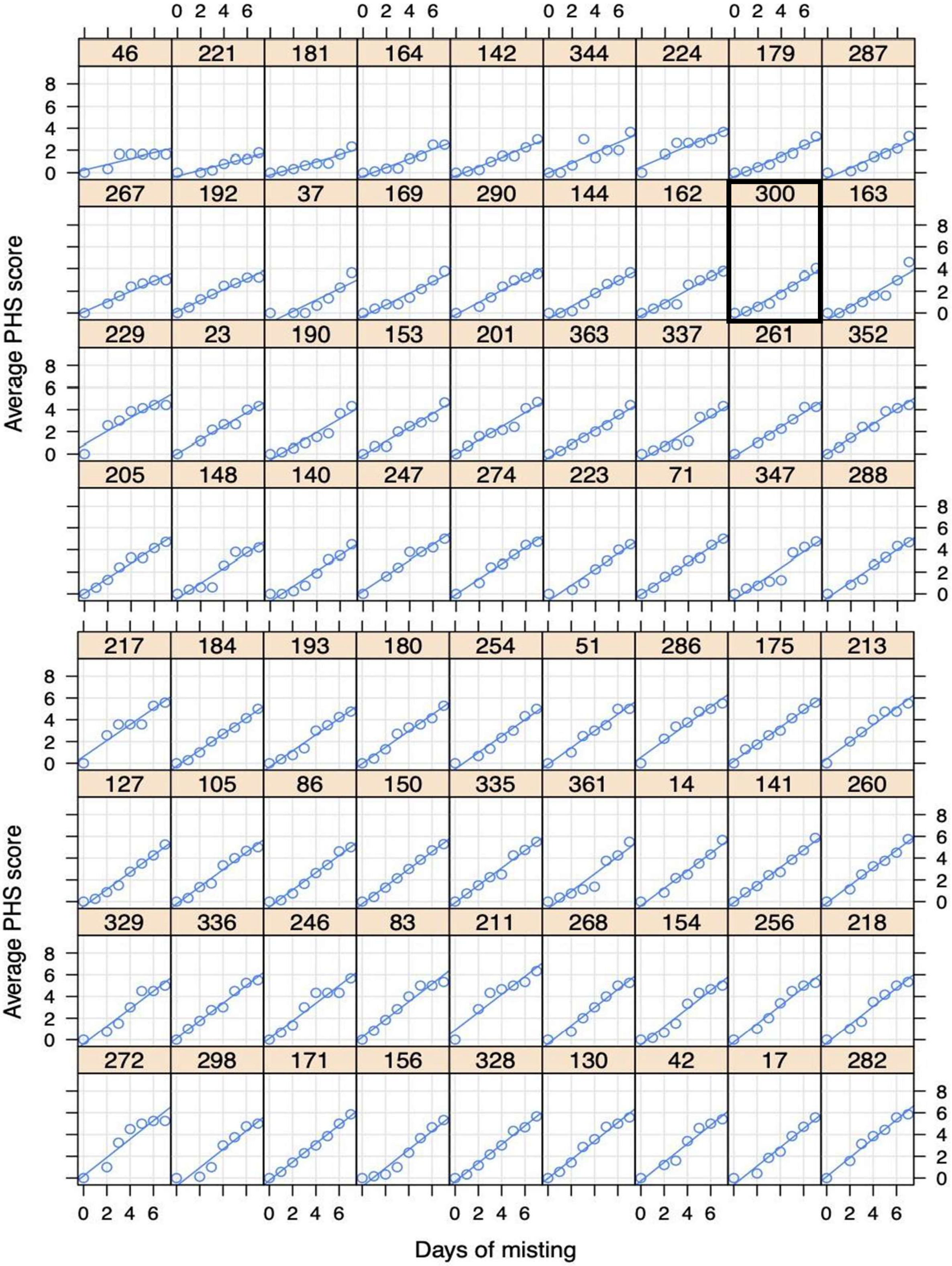

46
221
181
164
142
344
224
179
287
267
192
37
169
290
144
162
300
163
229
23
190
153
201
363
337
261
352
205
148
140
247
274
223
71
347
288
217
184
193
180
254
51
286
175
213
127
105
86
150
335
361
14
141
260
329
336
246
83
211
268
154
256
218
272
298
171
156
328
130
42
17
282
0 2 4 6
0 2 4 6 8
Average PHS score
Days of misting

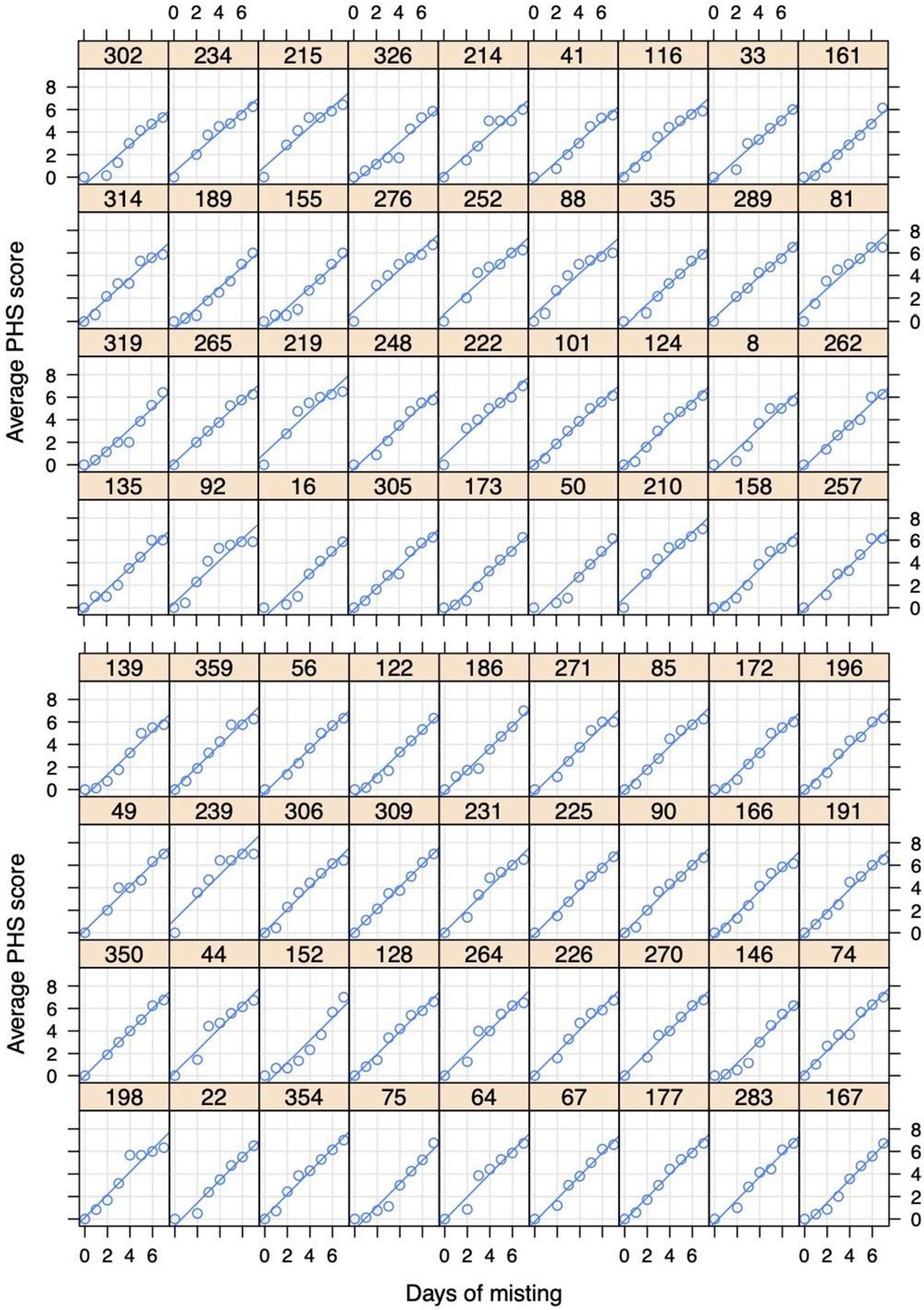

302 234 215 326 214 41 116 33 161
314 189 155 276 252 88 35 289 81
319 265 219 248 222 101 124 8 262
135 92 16 305 173 50 210 158 257
139 359 56 122 186 271 85 172 196
49 239 306 309 231 225 90 166 191
350 44 152 128 264 226 270 146 74
198 22 354 75 64 67 177 283 167
Average PHS score
Average PHS score
0 2 4 6
0 2 4 6 8
Days of misting

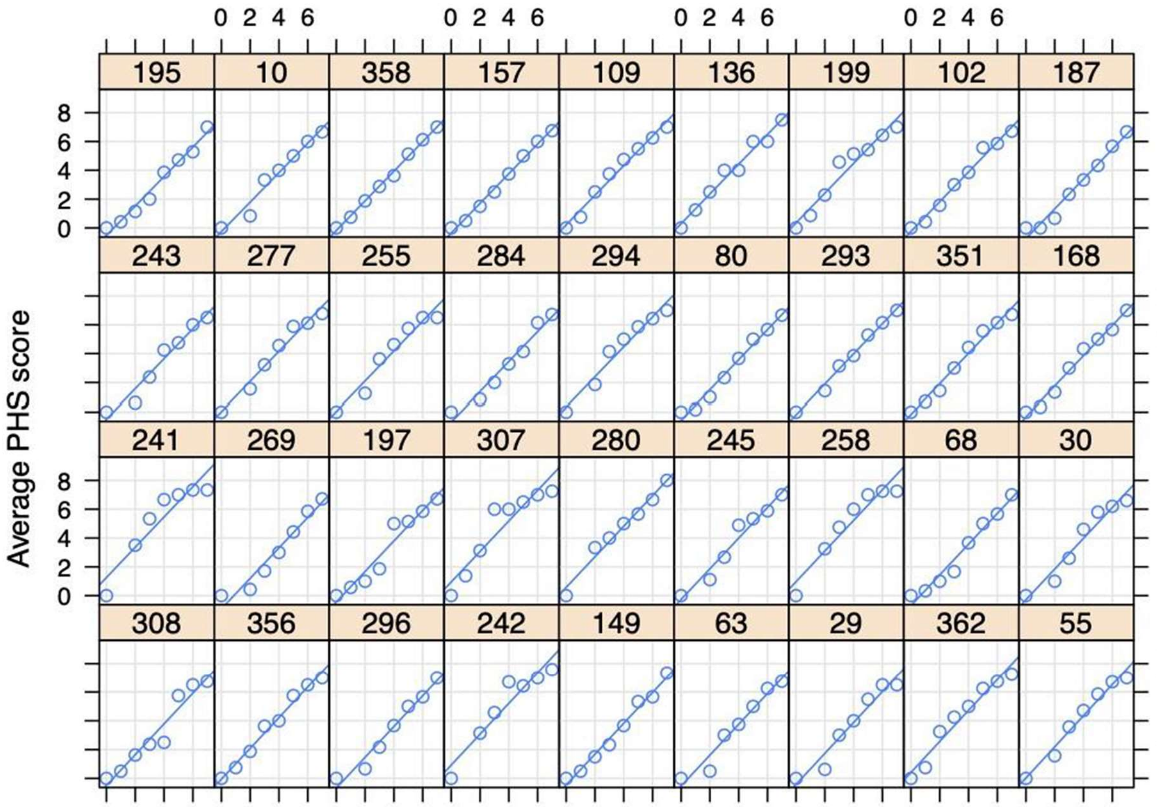
Average PHS score
195 10 358 157 109 136 199 102 187
243 277 255 284 294 80 293 351 168
241 269 197 307 280 245 258 68 30
308 356 296 242 149 63 29 362 55
0 2 4 6 8

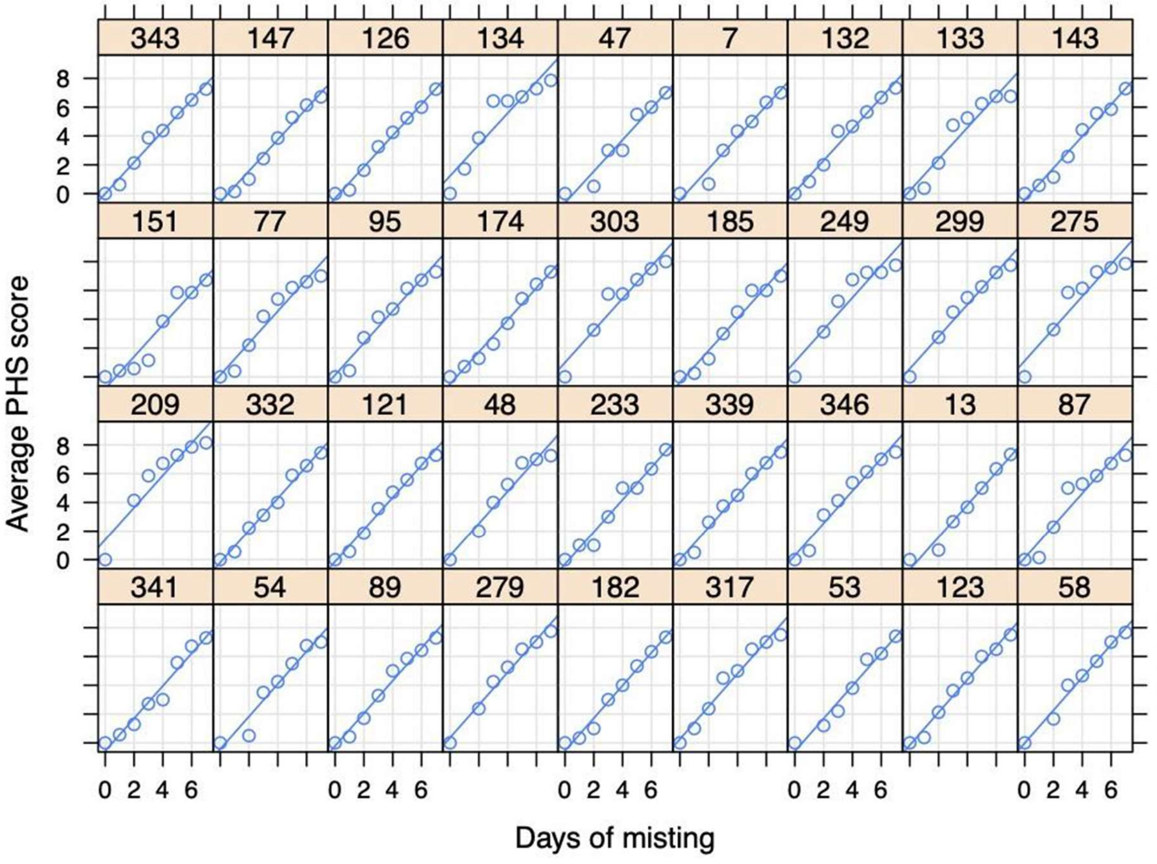
Average PHS score
343 147 126 134 47 7 132 133 143
151 77 95 174 303 185 249 299 275
209 332 121 48 233 339 346 13 87
341 54 89 279 182 317 53 123 58
0 2 4 6 8
Days of misting

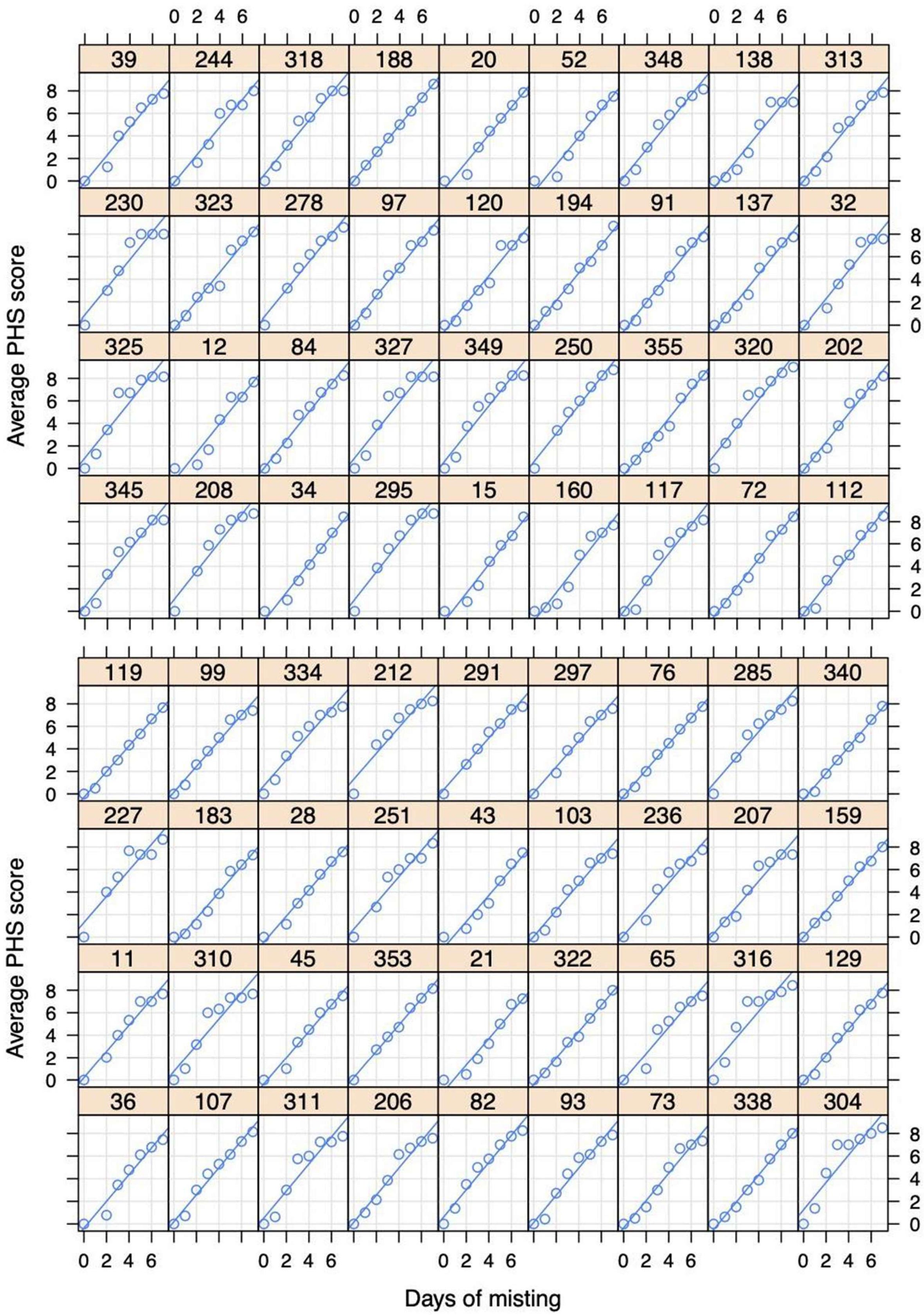

39 244 318 188 20 52 348 138 313
230 323 278 97 120 194 91 137 32
325 12 84 327 349 250 355 320 202
345 208 34 295 15 160 117 72 112
119 99 334 212 291 297 76 285 340
227 183 28 251 43 103 236 207 159
11 310 45 353 21 322 65 316 129
36 107 311 206 82 93 73 338 304
Average PHS score
Average PHS score
Days of misting

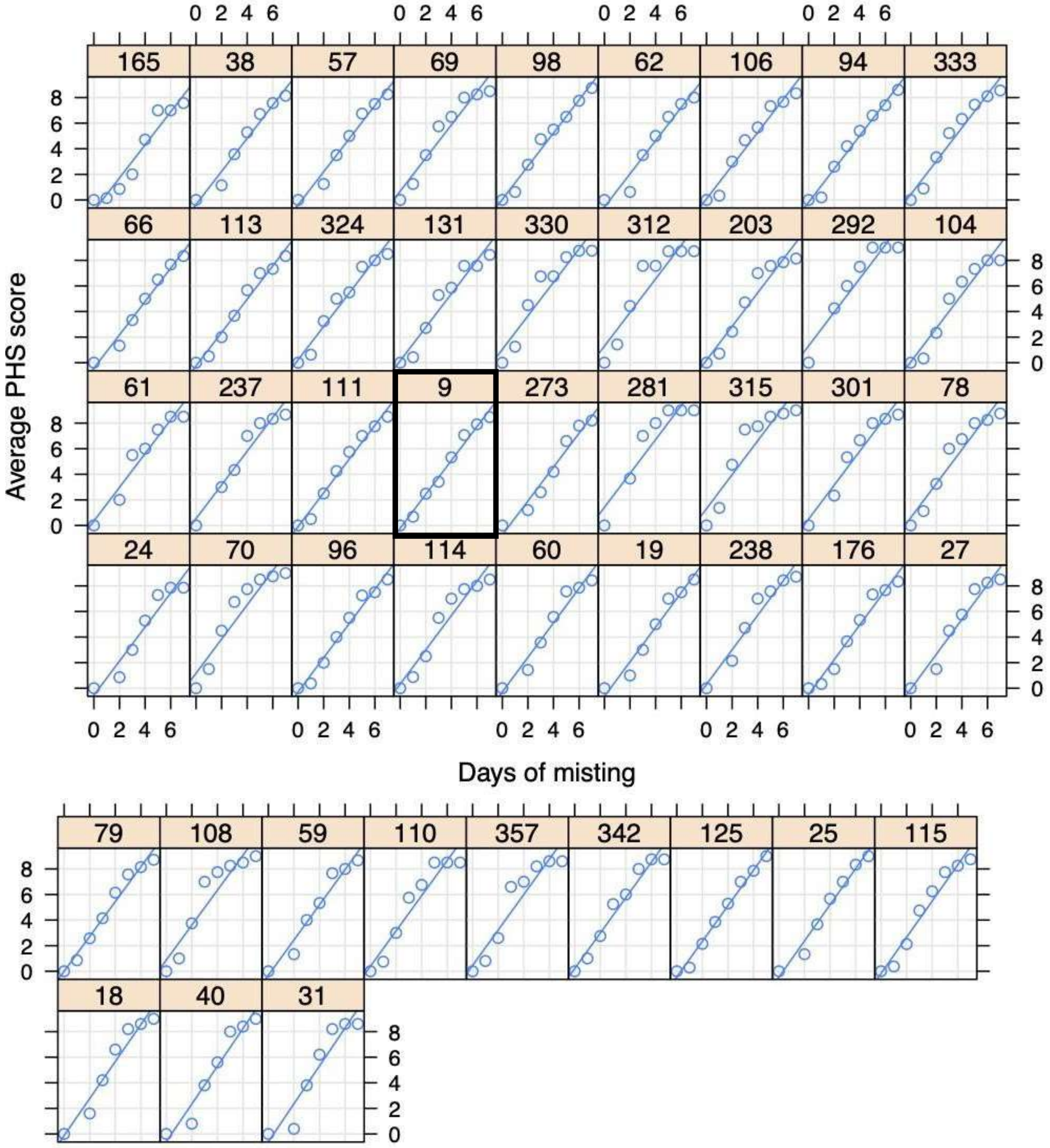

Figure S1: XY plots fitting a linear model for mean PHS scores (y axis) over 7 days of misting (x axis). Controls are bolded with a black line (ID 9 is PHS-susceptible, ID 300 is PHS-tolerant). Plots are shown ascendingly in respect to slope values, which reflect decreasing PHS tolerance. All genotypes include a value of 0 on day 0 to homogenize starting point for all lines.
