# Supplementary material for: Preharvest Sprouting in Quinoa: A New Screening Method Adapted to Panicles and GWAS Components
Source: Plants (Basel). 2024 May 8;13(10):1297. doi: 10.3390/plants13101297 (PMC11124833; doi:10.3390/plants13101297)
Supplement: Supplementary file 1 [file plants-13-01297-s001.zip › Quinoa_Fig S2.pptx]

## Slide 1
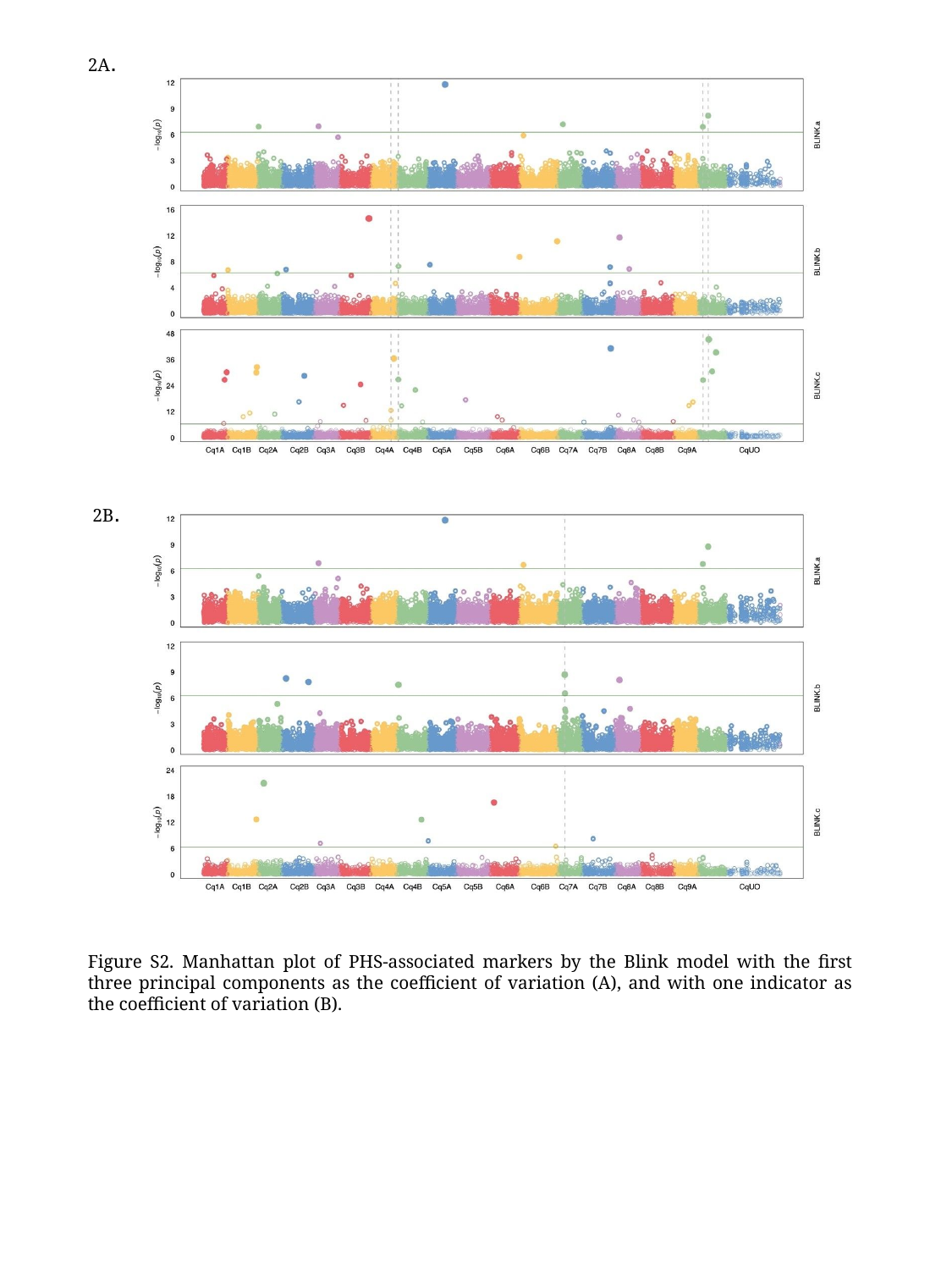

2A.
2B.
Figure S2. Manhattan plot of PHS-associated markers by the Blink model with the first three principal components as the coefficient of variation (A), and with one indicator as the coefficient of variation (B).
