# Supplementary material for: Preharvest Sprouting in Quinoa: A New Screening Method Adapted to Panicles and GWAS Components
Source: Plants (Basel). 2024 May 8;13(10):1297. doi: 10.3390/plants13101297 (PMC11124833; doi:10.3390/plants13101297)
Supplement: Supplementary file 1 [file plants-13-01297-s001.zip › Quinoa_Fig S3.pptx]

## Slide 1
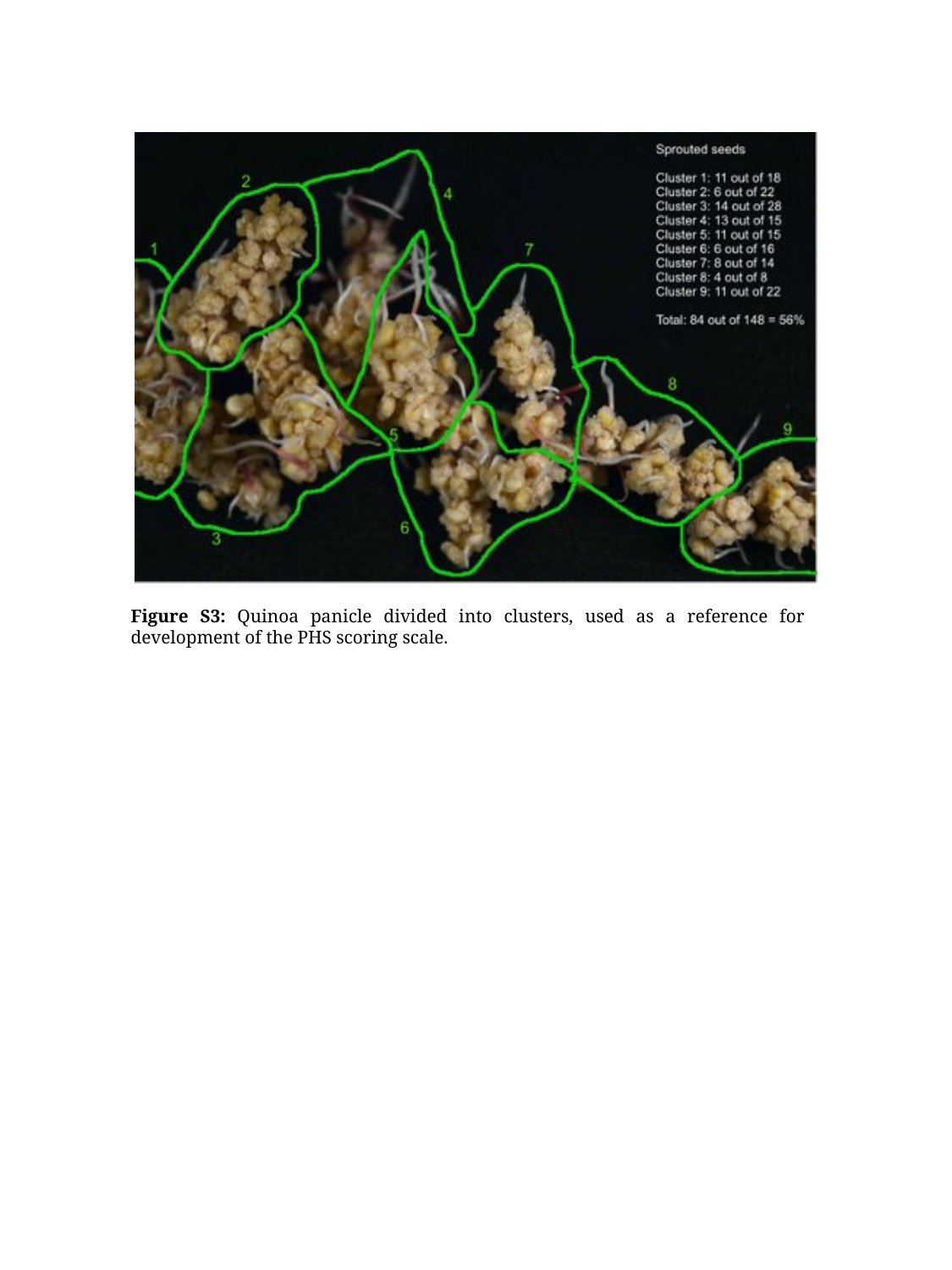

Figure S3: Quinoa panicle divided into clusters, used as a reference for development of the PHS scoring scale.
